# Advancing Lung Cancer Treatment: A Comprehensive Review of Photodynamic Therapy and Nanoparticle Applications

**DOI:** 10.3390/pharmaceutics17121579

**Published:** 2025-12-08

**Authors:** Andreea Moise-Crintea, Anne-Marie Constantin, Elena Mihaela Jianu, Ioana Maria Orlea, Minodora Manea, Roxana Oana Cojocariu, Rahela Carpa, Bogdan-Andrei Borlea, Cristina-Maria Boznea, Razvan Lucian Coseriu, Alina Sovrea

**Affiliations:** 1 Medical Biochemistry, Department of Molecular Sciences, Iuliu Hațieganu University of Medicine and Pharmacy, 400349 Cluj-Napoca, Romania; crintea.andreea@umfcluj.ro; 2 Histology, Department of Morphofunctional Sciences, Iuliu Haţieganu University of Medicine and Pharmacy, 400349 Cluj-Napoca, Romania; annemarie.chindris@umfcluj.ro (A.-M.C.); ioana.orlea@gmail.com (I.M.O.); alina.simona.sovrea01@gmail.com (A.S.); 3 Medical Psychology and Psychiatry, Department of Neurosciences, Iuliu Hațieganu University of Medicine and Pharmacy, 400023 Cluj-Napoca, Romania; mmanea@umfcluj.ro; 4 Faculty of Medicine and Biological Sciences, Ștefan cel Mare University, 720229 Suceava, Romania; roxana.cojocariu@usm.ro; 5 Department of Molecular Biology and Biotechnology, Faculty of Biology and Geology, Babes-Bolyai University, 400084 Cluj-Napoca, Romania; rahela.carpa@ubbcluj.ro; 6 Faculty of Medicine, Iuliu Hațieganu University of Medicine and Pharmacy, 400349 Cluj-Napoca, Romania; borlea.bogdan.andrei@elearn.umfcluj.ro (B.-A.B.); boznea.cristina.maria@elearn.umfcluj.ro (C.-M.B.); 7 Department of Medical Microbiology, “George Emil Palade” University of Medicine, Pharmacy, Science, and Technology, 540136 Târgu Mureș, Romania; razvan_coseriu@yahoo.com

**Keywords:** lung cancer, photodynamic therapy, nanoparticle applications, photosensitizers

## Abstract

Lung cancer remains a significant global health challenge. The high mortality rate is primarily caused by late diagnoses and the limitations of conventional therapies. Photodynamic therapy (PDT), which uses photosensitizing compounds, specific wavelengths of light, and oxygen to generate cytotoxic reactive oxygen species (ROS) that selectively destroy cancer cells, has emerged as a promising, minimally invasive alternative. Despite its advantages, traditional PDT has limitations. These include the limited penetration depth of light and the hypoxic nature of the tumor microenvironment. Nanotechnology has transformed PDT by enabling the precise delivery of photosensitizers, improving their stability, overcoming physiological barriers, and allowing for deeper tissue targeting. This review analyzes the molecular mechanisms of PDT, the evolution of photosensitizer and nanoparticle design, strategies to overcome PDT limitations, and the impact of the tumor microenvironment. Additionally, the potential of combining PDT with other cancer therapies, such as chemotherapy, immunotherapy, targeted therapy, radiotherapy, and gene therapy, is being investigated. While preclinical successes are remarkable, clinical implementation of nanoparticle-based PDT faces complex regulatory pathways, manufacturing scalability challenges, and the need for robust long-term safety data. Integrating artificial intelligence (AI) and biomarker discovery will accelerate the development of personalized treatments and usher in a new era of targeted oncology for lung cancer patients.

## 1. Introduction

Lung cancer remains the leading cause of cancer-related deaths worldwide [1]. Other risk factors include radon, asbestos, and air pollution [2]. The high mortality rate is due to the fact that those affected only develop the disease at an advanced stage. Only 26 percent are in stage I, and 8 percent are in stage II. In contrast, 28 percent are in stage III, while a significant percentage—around 38 percent—are already in stage IV, which is generally incurable and mainly treated palliatively [2].

The therapies for non-small cell lung cancer (NSCLC) vary depending on the stage of the disease. Stage I NSCLC is treated with surgery, while stages II and IIIA are treated with surgery followed by chemotherapy after the operation [3]. Radiotherapy, if tolerated, can be simultaneously with chemotherapy; otherwise, it is carried out sequentially. For non-resectable stage IIIA and IIIB cancers, chemotherapy and radiotherapy are administered simultaneously. Small cell lung cancer (SCLC) responds well to chemotherapy but often recurs [4,5]. The standard treatment is a combination of chemotherapy and radiotherapy. Recently, targeted therapies aimed at specific genetic mutations and immunotherapies have expanded treatment options for NSCLC [6,7].

Photodynamic therapy (PDT) is considered a promising alternative to chemotherapy and radiotherapy. Because PDT is less invasive than chemotherapy or radiotherapy, it was introduced in oncology and some other medical fields as early as 1963 with successful results [8,9]. The basic mechanism involves applying a photosensitizing compound extracted from tumor tissue. When the photosensitizing compound is excited by a specific wavelength of non-ionizing light in the presence of oxygen, it generates cytotoxic reactive oxygen species (ROS), which target the cancer cells [10]. Compared to standard therapies, PDT has the advantage of being able to cure tumors at an early stage, enabling long survival intervals and a good quality of life, even for patients who cannot undergo surgery. Other features that enhance its clinical profile include its ability to deliver treatment with minimal invasiveness and selectivity for diseased tissue, resulting in less destruction of healthy tissue [8,11,12].

Conventional lung cancer treatments have so far led to massive side effects and associated resistance, which underlines the need to develop a more effective, minimally invasive and targeted therapy [7]. Photodynamic therapy meets these requirements when applied with the help of nanotechnology, as it is precisely targeted and less toxic. This therefore represents a shift towards precision-guided oncology treatments that focus on patients’ quality of life by avoiding systemic toxicity. The focus is therefore on individualized, less harmful interventions that reflect the general trends in current oncology [12,13].

The integration of nanotechnology into PDT represents a major advance in the treatment of lung cancer. Nanoparticles are being developed to improve the delivery and efficacy of therapeutic agents. They increase the accumulation and stability of photosensitizers in tumor tissue and enable deeper penetration into the tumor, which is essential for effective PDT [11,12,13]. As advanced carriers, nanocarriers deliver photosensitizers to target cells with high precision, leading to better therapeutic outcomes and reduced systemic side effects. Improved drug loading efficiency and increased uptake by cancer cells contribute to a more effective and targeted treatment strategy [14,15,16]. Against this background, the aim of the present review is double. First, we summarise the molecular and cellular mechanisms of PDT and the evolution of photosensitisers and nanocarrier design with a specific focus on lung cancer. Second, we critically discuss how nanoparticle-based strategies are being used to overcome the main clinical limitations of PDT—namely limited light penetration, tumour hypoxia, and heterogeneous drug delivery—and how these advances translate into real-world protocols, patient selection, and outcomes. Compared with earlier reviews, we place particular emphasis on nanoparticle-enhanced PDT specifically in lung cancer, the interaction with the tumour microenvironment, and practical aspects of clinical translation, including regulatory and manufacturing issues and the emerging role of microfluidic/organ-on-a-chip models and artificial intelligence. Throughout the manuscript, our discussion focuses specifically on the application of PDT in lung cancer, rather than suggesting universal applicability to all malignancies.

## 2. Molecular and Cellular Mechanisms of Photodynamic Therapy

Photodynamic therapy works through a series of events at the molecular and cellular level that begin when a light-sensitive drug (photosensitizer) is activated by light. This process involves several steps and leads to the selective killing of cancer cells through the generation of ROS, which damage the cells [12,17].

The PDT process begins with administration of a light-sensitive drug (photosensitizer), either systemically or locally. These agents tend to accumulate in higher concentrations within tumors than in most normal tissues. Several properties of a photosensitizer—such as its chemical structure, lipophilicity, charge, and ability to bind plasma proteins and lipoproteins—strongly influence its biodistribution and subcellular localization [18,19]. Clinically useful and new generation photosensitizers are characterized by a high molar extinction coefficient in the red or near-infrared region (approximately 650–800 nm), where tissue absorption is relatively low and light can penetrate a few millimeters into tissue. A basic safety requirement is that the photosensitizer remains essentially non-toxic in the dark and becomes cytotoxic only after light activation [15,20,21]. A widely used example is porfimer sodium (Photofrin, which is a traditional PDT), which is typically activated with 630 nm laser light about 48 h after intravenous injection. Light is delivered through optical fibres with either a lens or a cylindrical diffuser tip to provide local or circumferential illumination. Newer photosensitizers have been developed with higher singlet-oxygen yields and absorption at longer wavelengths, which can improve treatment selectivity and depth of effect [22].

**Figure 1:**
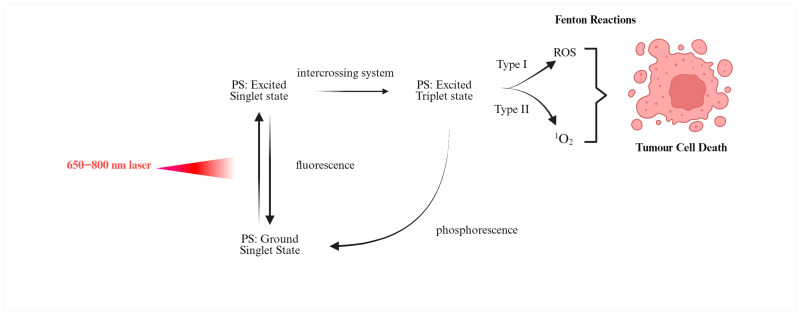
Schematic of the photodynamic reaction in PDT. After administration, the photosensitiser accumulates preferentially in tumour tissue. Illumination with light of an appropriate wavelength in the presence of oxygen excites the photosensitiser and leads to the formation of reactive oxygen species, mainly singlet oxygen. These short-lived species damage nearby cellular structures and ultimately result in tumour cell death. Created in BioRender. Elena Mihaela, J. (2025) https://BioRender.com/gz48iiq (accessed on 21 October 2025).

Photodynamic therapy kills cells primarily by generating ROS through a series of chemical reactions known as the photodynamic reaction. When the photosensitizer is exposed to light, it absorbs a photon and is excited to a singlet state. This activated photosensitizer can then release energy as fluorescence or undergo type I or type II photochemical reactions, both of which generate ROS [12,17,23]. In type I processes, electron or hydrogen transfer produces radical species that can subsequently react with oxygen, whereas in type II processes, energy transfer directly generates singlet oxygen. Singlet oxygen is highly reactive and has a very short lifetime on the order of tens of nanoseconds, which limits its diffusion radius to roughly 10–20 nm. This enables very localized destruction of cell parts. In contrast, in the type I PDT process, energy is transferred from the activated photosensitizer to nearby molecules such as nucleic acids, proteins and lipids, creating free radicals such as superoxide anions [23,24]. These radicals can further react with hydrogen peroxide or oxygen to generate other strong ROS, including hydroxyl radicals. The type II mechanism, which depends on oxygen, is particularly effective in superficial lesions, usually less than 1 cm deep, due to the rapid reactivity and short range of singlet oxygen [25,26]. In Figure 1 is emphasized the mechanism of photodynamic reaction during PDT.

Singlet oxygen kills cells through oxidation, which destroys important cell components such as amino acids, certain DNA bases and membrane lipids in the cell membranes and mitochondria. This damage triggers controlled cell death processes such as necrosis, apoptosis, paraptosis and autophagy. Which cell death pathway is activated depends on factors such as the chemical properties of the photosensitizer, its position in the cell and the amount of light used. Porfimer sodium, for example, targets both the cell membrane and the mitochondria [27,28]. Photosensitizers in mitochondria can trigger apoptosis at lower light intensities or necrosis and autophagy at higher intensities. Damage to the endoplasmic reticulum can lead to a strong release of calcium in the cell, which triggers necroptosis. High light intensity usually leads to necrosis, which involves rapid cell degradation and release of cell contents, while lower intensity causes apoptosis, a controlled form of cell death [29]. High doses can destroy these organisms and release their enzymes into the cytoplasm, leading to necrosis. The ability of PDT to kill cells depends largely on the production of singlet oxygen and the subsequent oxidation of cellular components, including DNA bases in the nucleus, the accumulation of misfolded proteins in the endoplasmic reticulum, oxidative stress in the mitochondria, and the oxidation of phospholipids that disrupt the plasma membrane [30,31,32]. By adjusting the PDT dose through the concentration of the photosensitizer and the light intensity, we can control which cell death pathways are activated. In practice, adjustments to PDT dose—through photosensitizer concentration and light fluence—do not selectively “choose” a single cell-death pathway, but they can shift the balance toward pathways such as apoptosis (favored by moderate oxidative stress) or necrosis (favored by overwhelming damage), while autophagy generally serves as an early protective mechanism rather than a terminal death route. Therefore, personalized dosing should not only focus on eliminating tumor cells, but also on choosing the type of cell death to improve the immune response or reduce inflammatory side effects [33].

Photodynamic therapy not only damages the cells directly, but also significantly impairs the blood supply to the tumor. Photosensitizers are deposited in the endothelial cells of the tumor’s blood vessels. When exposed to light, the photodynamic reaction causes necrosis in these vascular cells. This damage to the blood vessels leads to blockages, localized oxygen deprivation and the release of toxic substances such as thromboxane, cytokines and excess calcium. The destruction of the tumor’s blood vessels leads to a lack of nutrients and an accumulation of toxic waste products, which contributes to the death of the tumor cells. Blood vessel damage is an important factor that increases tumor cell death, not just a side effect. Therefore, effective PDT strategies should target both the cancer cells and the blood vessels of the tumor to maximize therapeutic efficacy [12,30,34,35].

An important aspect of PDT is its significant effect on the immune system. The release of cytokines, growth factors and proteins from PDT-damaged cells stimulates the body’s immune system, attracting neutrophils and macrophages to the treatment site, which contributes to the death of tumor cells [36]. These macrophages activate T helper lymphocytes, which in turn activate cytotoxic T lymphocytes responsible for tumor cell necrosis and apoptosis. This immune response is essential for long-term anti-tumor activity and helps to control metastasis and prevent tumor recurrence. PDT-induced cell death also triggers the release of inflammatory cytokines and damage-associated molecular patterns or alarmins into the bloodstream, resulting in a strong innate immune response that is further enhanced by adaptive immunity. Damage-associated molecular patterns such as calreticulin, high mobility group box 1 and ATP act as danger signals during tumor cell death, with calreticulin serving as an “eat me” signal for antigen-presenting cells and ATP as a “find me” signal for monocyte recruitment. The reactive oxygen species generated by PDT lead directly to the release of damage-associated molecular patterns and promote the maturation of dendritic cells and the activation of cytotoxic T lymphocytes [37,38,39]. This process transforms immunologically “cold” tumors, which do not respond to immune attack, into “hot” tumors, which are highly susceptible to immune-mediated destruction. PDT is therefore more than just a local treatment. It prepares the immune system to recognize and eliminate cancer cells, including those that have spread or reappeared. This makes PDT a valuable tool for systemic cancer control, especially in combination with immunotherapy. In addition, PDT promotes the growth of human mononuclear cells from the peripheral blood, further supporting its role in enhancing immune responses against cancer [40].

## 3. Advances in Photosensitizer Development and Formulation Strategies

The effectiveness of PDT depends to a large extent on the properties of the light-activated drug used, the photosensitizer. Over the years, the way these drugs are developed has changed significantly, from older versions that spread throughout the body to newer, more targeted options [8].

The development of photosensitizers is generally divided into different generations. This terminology is mainly historical and heuristic, but it is widely used in PDT literature and helps group agents according to typical clinical properties and design strategies rather than strict efficacy classes. First generation photosensitizers, such as porfimer sodium (Photofrin), were among the first to be approved for medical use and widely available. Although they worked, they often had disadvantages, in particular the problem that patients remained photosensitive for a long time as they remained in the body for a prolonged period [41]. The second-generation photosensitizers had better properties, including higher purity, improved production of singlet oxygen (a reactive form of oxygen) and activation with longer wavelengths of light, usually in the red or near-infrared (NIR) range (650–800 nm). This shift to longer wavelengths was important because it allowed the light to penetrate deeper into the tissue, making it possible to treat a wider variety of tumors. Examples of this are Verteporfin and Temoporfin [15]. Third-generation photosensitizers represent a significant advance that focuses on incorporating specific targeting approaches. These improved photosensitizing compounds are designed to increase their attraction to tumor tissue while reducing their accumulation in healthy tissues. This is often achieved by binding the photosensitizer to targeting molecules such as antibodies that recognize specific markers on tumor cells, allowing selective binding and localization to the diseased site [13,42]. Other strategies use receptors that are found in larger quantities on the surface of tumor cells, such as the low-density lipoprotein receptor and the folate receptor. Tiny carrier systems such as liposomes or targeted nanoparticles are also used to deliver the photosensitizing compound specifically to the tumor. This targeted approach not only improves efficacy, but also helps the drug to be cleared from the body more quickly. Studies have shown that photosensitizers bound to fragments of single-chain monoclonal antibodies is removed more effectively [43,44].

**Figure 2:**
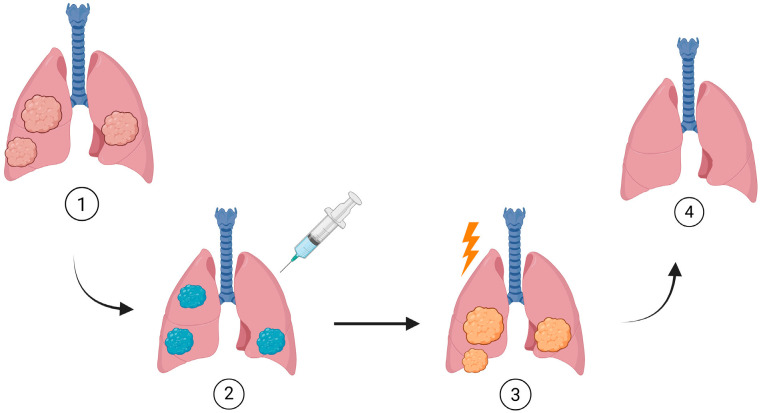
Photodynamic therapy workflow for early-stage central lung cancer. (1) An early-stage endobronchial tumor (e.g., CIS or small T1 lesion) is detected, typically by bronchoscopy and imaging. (2) After diagnosis, a photosensitizer is administered intravenously and allowed to accumulate preferentially in the tumor. (3) At the planned time point, therapeutic bronchoscopy is performed, and light of a specific wavelength is delivered to the lesion via optical fibers, activating the photosensitizer within the bronchial lumen. The yellow symbol represents the light of the specific wavelenght delivered as treatment. (4) Activated photosensitizer generates ROS, which induce cytotoxic effects and lead mainly to apoptotic or necrotic death of the tumor cells. This schematic illustrates the treatment sequence in clinically diagnosed patients and is not intended to represent a population-wide screening procedure. Created in BioRender. Elena Mihaela, J. (2025) https://BioRender.com/7oolrc2 (accessed on 21 October 2025).

According to Figure 2, improving the photosensitive properties of photosensitizing compound is a constant focus in their development. Desirable properties include strong light absorption characterized by a high absorption coefficient, especially in the red or NIR spectrum, which is essential for deeper tissue penetration. Second-generation photosensitizing compound offer a higher yield of singlet oxygen, which directly leads to higher cell-killing efficiency. Examination of certain photosensitizing compound illustrates these advances [45], for example, a Photofrin II (sodium porphimer), a type II PDT photosensitizer that can penetrate deep into tissue (absorption at ~630 nm). However, its complex processing by the body leads to a long elimination half-life of over 100 h, resulting in prolonged photosensitization (4–6 weeks after administration). Accumulation in tumors is primarily passive, leading to accumulation in normal tissues, particularly the skin [46].

Verteporfin (Visudyne), also a type II photosensitizer, its formulation in liposomes increases its solubility and allows for rapid elimination from the body (half-life = 5–6 h), significantly reducing prolonged photosensitization compared to Photofrin II. However, it is less stable under light and may undergo self-polymerization or photobleaching, which may reduce its efficacy [47].

5-aminolevulinic acid activity is achieved by conversion to protoporphyrin IX, a highly potent photosensitizer, by enzymes involved in the heme production pathway. Protoporphyrin IX selectively accumulates in cancerous or abnormal tissues. Its photosensitizing effect lasts for 14 to 18 h. Limitations include the uneven distribution of photosensitizers in tumor tissue and the limited depth to which the laser can penetrate [48].

Temoporfin (mTHPC, Foscan), a potent second-generation photosensitizer with a strong absorption peak in the red spectral region (~652 nm), allowing deeper penetration into the skin and efficient production of singlet oxygen. Its high affinity for lipids promotes preferential accumulation in malignant tissue, but also leads to extensive binding to proteins in the blood and potential off-target phototoxicity and photosensitization of the skin [49].

Photocyanin, a new phthalocyanine-based photosensitizer with a high singlet oxygen quantum yield (ΦΔ = 0.53) and strong absorption at 670 nm for enhanced tissue penetration has a well-defined structure, low dose requirement, minimal toxicity in the dark and minimal phototoxicity to the skin. Despite these advantages, the efficacy of PDT is still limited by its relatively low tissue penetration [50].

The natural aversion of many effective photosensitizers to water poses a challenge. While hydrophobic photosensitizing compounds have a better tumor-to-normal tissue ratio (between 7:1 and 8:1) compared to their hydrophilic counterparts (typically 2:1), this property causes them to clump together in an aqueous environment, making them unsuitable for direct injection into the bloodstream. This highlights a fundamental problem in the development of photosensitizing drugs [51]. While the hydrophobic properties are advantageous for passive accumulation in tumors through mechanisms such as the enhanced permeability and retention (EPR) effect and pH-induced diffusion, this poses a significant challenge for systemic delivery and formulation stability. Nanocarriers are therefore not simply transport vehicles, but essential solubilizing and stabilizing platforms that unlock the therapeutic potential of these intrinsically effective but problematic hydrophobic photosensitizers. This underlines the crucial role of nanomedicine in the formulation of photosensitizing compounds [52,53,54].

Strategies for improved stability and delivery are crucial for the clinical translation of therapies. Nanoparticles play a central role in this process as they increase the accumulation of photosensitizing compounds in tumor tissue, improve their stability and enable deeper penetration into the tumor. They effectively overcome problems such as poor solubility, low tumor specificity and clumping in aqueous environments [55]. Nanoemulsions, for example, have been shown to overcome these obstacles by enabling deeper penetration and more uniform distribution of photosensitisers in tumor tissue. They offer superior stability (up to 12 months compared to 1–3 months for liposomes) and efficiently encapsulate poorly water-soluble (hydrophobic) photosensitizing compounds. Nanoemulsions promote cellular uptake through mechanisms such as fusion with the cell membrane and pinocytosis. Controlled extracellular release can further enhance uptake by reducing self-aggregation. They may also contain up-conversion nanoparticles for improved light penetration and oxygen-generating compounds to increase oxygen levels in tumors [56]. Natural biomacromolecules such as human serum albumin are of key importance due to their biocompatibility, biodegradability, prolonged circulation and targeting capabilities as protein carriers. Human serum albumin significantly improves the solubility, stability and tumor targeting ability of poorly water-soluble photosensitizers by utilising multiple ligand-binding domains and EPR-independent tumor targeting. Other nanocarrier-mediated delivery systems, such as liposomal encapsulation and polymeric micelles, also address solubility issues, optimise pharmacokinetics and improve tumor-selective accumulation of hydrophobic photosensitizing compounds [57,58].

To improve the therapeutic efficacy of PDT while limiting unwanted side effects, strategies for selective targeting of the tumor are crucial. This discrepancy emphasises the urgent need for improved selectivity. Targeted interventions aim to increase the tropism of photosensitizing compounds for tumor tissue and thereby reduce damage to healthy cells. For this purpose, targeted components such as antibodies directed against tumor-associated antigens are often conjugated directly to the photosensitizing molecule [59]. Another effective approach is to exploit receptor-positive sites, especially those that are overexpressed on the surface of tumor cells, such as LDL and folate receptors. In addition, the acidic environment that is characteristic of tumor tissue can be exploited. The increased hydrophobicity of photosensitizing compound carrying carboxyl groups under acidic conditions favours their preferential localisation within tumor cells by passive diffusion through the plasma membrane. It is assumed that lipophilic photosensitisers modified with cationic functions selectively accumulate in the mitochondria, a central organelle in the initiation of apoptosis. It is clear that the discipline is moving towards sophisticated, “smart” delivery methods that not only accumulate in the tumor but also selectively interact with malignant cells or their characteristic components of the microenvironment [60,61]. This active targeting is crucial for optimising therapeutic effect, minimising off-target toxicity (e.g., skin photosensitivity) and enabling personalised therapeutic paradigms targeting specific biomarkers. This development points to a future where the delivery of photosensitizing compound achieves the highest level of precision, analogous to a “guided missile” delivery system rather than a less targeted “shotgun” approach [62].

Table 1 summarizes recent advances in photosensitizer development and formulation strategies.

## 4. Enhanced Photodynamic Therapy in Lung Cancer Through Nanoparticle Technologies

Nanoparticle technologies are revolutionising PDT for lung cancer by enhancing the delivery of photosensitizers to better target the tumor and overcome treatment challenges. The design of these tiny platforms is critical as their specific physical and chemical properties influence their functionality in the body and their effectiveness in treating disease [11,13,62].

Nanoparticles are typically between 1 and 100 nm in size, which is compatible with many structures in the body and allows them to penetrate biological systems. They are usually composed of organic polymers, lipids, proteins, or inorganic materials such as metals, metal oxides, or silica. Their ability to mix with both water and fat is crucial as it allows nanoparticle–photosensitizer complexes to circulate in the bloodstream without being rapidly cleared or degraded. Other properties such as shape, composition, the amount of drug they can carry, the way they release drugs and their surface properties also impact on their functionality. Careful control of these properties during manufacture is key to ensuring that the drug is loaded correctly and released at the right time [63,64,65].

A higher proportion of nanoparticle accumulation in the tumor environment compared to normal tissue is achieved by two primary mechanisms: passive and active targeting [66].

Passive targeting mechanism takes advantage of the unique pathophysiological properties of solid tumors, which are summarised under the term EPR effect. The blood vessels of tumors are often structurally abnormal and characterised by disordered, loosely connected, branched, overlapping or sprouting endothelial cells, making them “leaky”. At the same time, solid tumors typically have poorly developed or defective lymphatic drainage systems. This combination allows macromolecules and nanoparticles to extravasate from the leaky vasculature and be retained in the tumor interstitium, leading to preferential accumulation. The components of the extracellular matrix (ECM) in the tumor stroma, such as collagen, elastin and hyaluronan, can further aid drug retention, with some photoactive porphyrin derivatives preferentially interacting with collagen. In addition, the acidic microenvironment in tumors resulting from increased anaerobic glycolysis may enhance the hydrophobic properties of photosensitizing compounds with carboxyl groups and promote their preferential diffusion into tumor cells [67,68,69].

Nanoparticles are provided with specific molecules that bind to receptors or antigens that are mainly located on tumor cells or their blood vessels, but not on healthy cells. This can include the binding of antibodies to tumor antigens or the use of receptors such as LDL and folic acid receptors. After the nanoparticles first accumulate in the tumor, this targeted binding helps them to accumulate more in the tumor, often increasing the amount of photosensitiser in the cells [54,70].

Nanoparticles used in conjunction with PDT for NSCLC can be broadly categorised into organic and inorganic types, each with unique properties and methods of synthesis [71].

### 4.1. Organic Nanoparticles

This category includes materials such as graphene quantum dots, which have been investigated for their ability to generate singlet oxygen and improve PDT efficacy in NSCLC. Carbon nitride–based nanoparticles decorated with carbon dots have been reported to enhance ROS generation under light irradiation, in part by photocatalytically converting endogenous species such as H_2_O_2_ into molecular oxygen, thereby improving local oxygen availability, thereby enhancing PDT efficacy under both hypoxic and normoxic conditions [72]. Glucose-derived nanoparticles induce rapid cell death in lung cancer cells upon activation by increasing heat shock proteins and DNA damage, while being non-cytotoxic to primary human macrophages and susceptible to biodegradation by human neutrophils [73].

### 4.2. Polymeric Nanoparticles

These versatile platforms include hyaluronic acid ceramides, which solve problems such as poor solubility, low tumor specificity and aggregation of photosensitizing compound in an aqueous environment, thus significantly improve PDT. Conjugated polymers, known for their efficient fluorescence and strong light absorption, are used to improve singlet oxygen generation [74]. Dextran-based micelles have been developed to integrate photosensitizing and chemiluminescent agents to enable in situ light generation for PDT by chemiluminescence resonance energy transfer [75]. Polymeric nanocarriers generally offer advantages such as biocompatibility, biodegradability, high drug loading, sustained drug release, scalability, low batch-to-batch variability, low-cost production and easy tunability due to their coreshell structure [76]. Elastin-like polypeptides are particularly promising biomaterials due to their excellent biocompatibility, biodegradability and the precise tunability of their physicochemical properties, including molecular weight and hydrophobicity, by modifying their amino acid sequence [77].

### 4.3. Porphyrin-Based Nanoparticles

These are self-assembled from porphyrin derivatives and offer the decisive advantage that they target specific receptors that are expressed on lung cancer cells. For example, porphyrin high-density lipoprotein nanoparticles target the class B type I scavenger receptor on NSCLC cells, promoting selective accumulation and photoactivation in tumors [78]. Porphyrin-cholesterol conjugates can assemble into nanoparticles without surfactants or amphiphilic polymers, which accumulate at the tumor site and induce immunogenic cell death by stimulating and recruiting antigen presenting cells to mature and activate T cells. Porphyrin lipid nanoparticles (porphysomes) targeting folic acid receptor 1 accumulate in lung tumors and lead to a significantly improved contrast between diseased and normal tissue, successfully inhibiting tumor cell proliferation and activating tumor cell apoptosis. Covalent organic frameworks, formed by a bottom-up approach from molecular building blocks, provide high permanent porosity that promotes both the diffusion of oxygen and the release of ROS into cells, combined with excellent photostability and biocompatibility [79,80,81].

Nanoparticles based on boron-dipyrromethene are characterised by an acceptor-donor-acceptor structure, exhibit high photostability and a high molar extinction coefficient, ensuring robust and sustained production of ROS. They can be used for the hierarchical degradation of nanomolecules in response to the acidic tumor environment [82].

### 4.4. Inorganic Nanoparticles

General synthesis methods for inorganic nanoparticles include physical “top-down” approaches such as ultrasound, microwave irradiation, evaporation condensation and laser ablation, which reduce material size. Chemical approaches include metallic intermediates, stabilising agents and reducing agents. Increasingly, environmentally friendly biological methods, also known as “green synthesis”, are also being used, in which plants, fungi, microorganisms or their by-products such as proteins and lipids are used to produce nanomaterials [83,84].

### 4.5. Metal-Based Nanoparticles

Metal-based nanoparticles (e.g., gold (Au nanoparticles) or silver (Ag nanoparticles)) have become established in medicine since 2014 due to their versatility in terms of size and shape as well as their long duration of action. Gold nanoprisms, triangular prismatic Au nanopartciles, have been developed for NSCLC and conjugated with chlorin e6 (Ce6) and rogrammed death-ligand 1 peptides to increase targeting efficiency. They show significant suppression of tumor growth by combined photothermal therapy and PDT, with the gold nanoprisms facilitating photothermal conversion and Ce6 acting as a photosensitiser. A photoactive curcumin-silver nanoparticle-polymer conjugate was developed to enhance lung cancer PDT by targeting both lung cancer cells and lung cancer stem cells. Curcumin itself is typically activated by blue light in the 420–480 nm range, and this conjugation strategy preserves its photo-reactivity while improving stability and cellular uptake [85,86,87].

Hybrid nanostructures that incorporate magnetic nanoparticles (MNPs) have attracted increasing attention in recent years, particularly for their potential to enhance photodynamic therapy (PDT) [88]. By combining a magnetic core—most often iron oxide—with photosensitisers, these systems bring several functions together in a single platform. In addition to carrying the therapeutic agent, they can be used for imaging, magnetic guidance, and for generating complementary photothermal effects, making them appealing as multifunctional tools for cancer treatment [88,89]. The ability to steer these particles with an external magnetic field offers a way to concentrate the therapeutic payload within selected lung regions, which could help improve treatment precision while limiting unwanted exposure of surrounding tissues [90,91].

Beyond their targeting capabilities, magnetic nanoparticles can also serve as contrast agents for MRI and can generate localised heat when exposed to an alternating magnetic field [90]. Magnetic targeting is achieved by applying an external magnetic field to the tumour region, which attracts nanoparticles containing magnetic iron oxide cores. Alternating magnetic fields can induce localized heating through Néel and Brownian relaxation mechanisms, enabling magnetic hyperthermia in vivo when combined with PDT.NIR When this heating effect is combined with the reactive oxygen species produced during PDT, the two mechanisms may work together to improve tumour destruction [92]. Although most magnetic–PDT hybrid platforms are still at an early, experimental stage, they highlight the growing interest in “theranostic” systems—materials that allow imaging, targeted delivery and therapy in a single construct. Such approaches could eventually support more personalised strategies for managing lung cancer and other solid tumours [93,94,95].

Recent studies have started to explore different ways of designing these hybrid materials, often using advanced synthesis methods to fine-tune their optical, magnetic and chemical properties [96]. Some of the most promising work involves near-infrared responsive agents that allow real-time imaging and treatment within deeper tissues [94]. Other research is focused on systems that deliberately combine photothermal and photodynamic processes to produce stronger antitumour effects [97,98]. Together, these advances underline the versatility of magnetic hybrid nanomaterials and their potential role in shaping future PDT-based cancer therapies.

### 4.6. Metal Oxide Nanoparticles

Iron oxide nanoparticles loaded with indocyanine green and modified with hyaluronic acid have shown significant suppression of tumor growth in NSCLC models. Other metal oxide nanoparticles, in particular zinc oxide, are known for their biocompatibility and stability and have the ability to generate ROS, which distinguishes them from other inorganic nanoparticles [74,99,100].

### 4.7. Meosoporous Silica Nanoparticles

These are very effective for the precise administration of medication. Mesoporous silica nanoparticles, produced by polymerisation of silica, offers desirable properties such as a large surface-to-volume ratio, automatic drug release and the ability to incorporate various functional ligands. Their pore size can be customised to increase photosensitizing compound loading capacity. Mesoporous silica nanoparticles doped with anthraquinone for NSCLC have shown significant cytotoxicity by promoting cell apoptosis through both type I and type II mechanisms. Surface functionalisation with polyethylene glycol increases stability and prolongs circulation time, while the addition of folic acid enables active targeting of overexpressed folic acid receptors on cancer cells [101,102,103].

### 4.8. Quantum Dots (QDs)

These nanoscale semiconductor crystals have unique optical properties, including high emission quantum yield and easy surface modification, making them promising nanocarriers. Quantum dots can be engineered to absorb and emit light at specific wavelengths, facilitating activation by NIR light, which penetrates deeper into tissue compared to visible light, making them particularly effective for the treatment of deep-seated tumors. The utility of NIR lasers depends entirely on the availability of photosensitizers with strong absorption in the NIR range; without such agents, NIR illumination offers no therapeutic benefit [104,105]. Cobalt ferrite quantum dots have been developed for synergistic photothermal and photo dynamic therapy of NSCLC by significantly enhancing ROS generation and inducing apoptosis. Up-conversion nanoparticles are a special type of QD that convert NIR light into UV-visible light, which then activates photosensitisers and enables deeper penetration [106].

### 4.9. Biomimetic Nanoparticles

Biomimetic nanoparticles have quickly become one of the more intriguing developments in photodynamic therapy (PDT). The basic idea is straightforward: by combining a synthetic nanoparticle core with natural biological membranes or components, these systems are able to take advantage of the body’s own cellular machinery [107]. For example, when nanoparticles are coated with red blood cell, platelet, or even cancer-cell membranes, they tend to circulate for longer periods and are less likely to be flagged and cleared by the immune system [107]. These coatings also help the particles follow natural “homing” signals, improving their ability to reach tumor tissue or inflamed sites. Similar behaviour has been observed with exosomes and other extracellular vesicles, which has encouraged interest in using these naturally derived structures as carriers for photosensitizers in PDT [107,108].

One of the main advantages of these biomimetic systems is that they can deliver photosensitizers together with other therapeutic agents—such as immunomodulators or chemotherapeutics—while retaining a surface that the body recognises as “self.” This helps reduce unwanted uptake in healthy tissues and improves overall biocompatibility [109]. Early preclinical studies in lung cancer models are particularly promising, showing that these membrane-coated nanoparticles can accumulate more effectively inside tumors and, in some cases, strengthen both local tumor control and broader immune responses [110]. Exosomes, which naturally carry proteins and genetic material, have also gained attention because they are minimally immunogenic and able to cross biological barriers, making them appealing vehicles for drug or gene delivery [109].

Recent work has focused on improving exosome production and tailoring their contents—for instance, by loading them with photosensitizers or additional targeting molecules—to create more precise PDT delivery platforms [111]. There is also growing evidence that exosomes released from PDT-treated tumor cells can stimulate antitumor immune activity, adding an immunological dimension to their therapeutic potential [107,112].

### 4.10. Microfluidic and Organ-on-Chip Platforms

Microfluidic and organ-on-a-chip platforms are becoming important tools for improving both the synthesis and testing of photodynamic therapy (PDT) agents. Microfluidic reactors offer tight control over mixing, temperature and reaction time, which helps produce more consistent nanoparticle formulations than conventional batch methods and supports better scalability [113,114].

At the same time, organ-on-a-chip and tumour-on-a-chip models provide in vitro environments that more closely resemble human physiology, including microvascular flow, oxygen gradients and complex tissue architecture [115]. These systems have been used to study how nanoparticles and photosensitizers penetrate, accumulate and clear within tumour-like structures, and to replicate clinically relevant barriers such as the blood–brain and blood–tumour barriers [116,117]

Such platforms are also valuable for assessing light delivery, oxygen dynamics and treatment-related vascular or immune responses in a controlled setting, helping refine nanoparticle design and dosing strategies before moving into animal studies [118,119]. More recently, chip-based systems have been combined with high-throughput approaches to simulate drug distribution and therapeutic responses across different tumour architectures, offering a practical way to optimise PDT protocols [120].

Optimising the properties of nanoparticles is crucial to improving their performance. For example, larger polymeric nanocarriers tend to remain in the bloodstream longer and accumulate more in tumors. Research on elastin-like polypeptides suggests that larger, micelle forming versions penetrate tumors faster and deliver a higher drug dose than smaller or free drugs. Smaller elastin-like polypeptides tend to dissolve quickly, making them less effective. This means that it is important to choose the right size and the right surface properties. The field is now focusing on developing nanoparticles that are carefully tailored to each tumor type and delivery method, rather than using a single design for all cases. This requires advanced technology [61,73,121].

Modifying the surface of nanoparticles is another important way to improve their properties. Adding targeting molecules, helps the nanoparticles to adhere to tumor cells and penetrate them more easily. Coating nanoparticles with polyethylene glycol (PEG) makes them more stable, keeps them in the bloodstream longer and helps prevent immune reactions. Folic acid is often used to enable nanoparticles to penetrate cancer cells, which have a high proportion of folic acid receptors. The pore size of nanoparticles of mesoporous silica can be adjusted to accommodate more photosensitisers [61,122,123]. Nanoparticles can also be designed to release their active ingredient only when triggered by certain conditions in the tumor, such as changes in pH or exposure to light. This means that the active substance is only activated in the tumor and not in healthy tissue. For example, acid sensitive linkers can accelerate the release of drugs in the acidic environment of tumors. Nanoparticles are becoming more and more advanced and are able to fulfill several functions simultaneously in the tumor. This ability to overcome multiple challenges simultaneously, such as oxygen starvation, light delivery, targeting and imaging, significantly increases their potential [124,125,126,127].

## 5. Overcoming Inherent Limitations of PDT

Photodynamic therapy holds great potential, but has two major problems: the low oxygen content in tumors and the short range of the light in body tissue. Researchers are developing new nanoparticles and methods of light transmission to solve these problems and make PDT accessible to a wider audience [12,42].

In most cases, visible light reaches no deeper than about 5–10 mm, which means that standard PDT can only be applied reliably to surface tumors or to lesions that physicians can reach with an endoscope [128]. This basic physical barrier becomes particularly problematic when dealing with cancers that grow deeper in the body, such as many forms of lung cancer. As a result, much of the recent research in the field has focused on finding new ways to deliver light more effectively to these hard-to-reach areas [129,130]. Technologies like NIR lasers and specially designed fiber-optic systems are now being explored as possible ways to deliver light further into tissue and activate photosensitizers that would otherwise remain inaccessible [131,132]. Current investigational light-delivery systems include miniaturized fiber-optic diffusers and bronchoscope-compatible cylindrical fibers designed to illuminate airway lesions more uniformly [133].

To push PDT beyond its traditional limits, several promising strategies have emerged. One approach that has attracted a great deal of interest involves the use of upconversion nanoparticles (UCNPs), which are capable of absorbing NIR light and converting it into visible wavelengths that can activate photosensitizers deeper inside the tumor [129,134]. This helps bypass the usual problems caused by tissue absorption and scattering, which are the main reasons light fails to penetrate very far. Another inventive method uses Cerenkov radiation—light produced by certain radionuclides—to generate illumination directly inside the tumor, eliminating the need to push light in from the outside. Cerenkov radiation, generated during radionuclide decay, produces only low levels of light and requires exposure to ionizing radiation. Its potential use in PDT remains experimental and is limited by low light intensity and safety considerations [130,135].

Alongside these advances in light delivery, nanotechnology has opened up new options for improving both the precision and effectiveness of PDT. Many nanoparticle-based carrier systems can deliver photosensitizers straight into cancer cells, limiting exposure to healthy tissue and allowing for more focused treatment [136]. Careful control of light dosimetry—essentially, how much light is given and how it is distributed—can further improve outcomes, especially in tumors that are uneven in size or density [131,137,138]. Fractionated light schedules, in which illumination is delivered in intervals rather than all at once, have also been shown to help by giving the tissue time to reoxygenate, a particularly useful feature when dealing with large or densely packed tumors [137,139].

A major problem for PDT is that tumors often have low oxygen levels. PDT relies on oxygen to generate ROS. However, the blood vessels in tumors are often disorganised, leading to areas of low oxygen and making PDT less effective. Nanoparticles offer innovative solutions, such as the integration of components that produce oxygen, split water or improve blood flow. These approaches exploit the weakness of a tumor to achieve a treatment benefit. Some important nanoparticle strategies to combat tumor hypoxia are detailed below [140,141]:

### 5.1. Oxygen-Generating Nanoparticles

These nanoparticles are designed to produce oxygen directly in the tumor. Some contain catalase-like components that generate oxygen on the spot during PDT and can thus overcome tumor hypoxia. Manganese dioxide nanoparticles, which degrade hydrogen peroxide to generate O_2_ and improve T1-weighted magnetic resonance imaging, are primarily used for this purpose [142,143]. However, endogenous H_2_O_2_ levels vary widely between tumor types and may be insufficient in some settings, so these strategies remain largely at the preclinical proof-of-concept stage. Another example is carbon-dot-decorated carbon nitride nanoparticles, which improve intracellular oxygen concentration and ROS generation by photolysis upon light irradiation and increase the efficacy of PDT under both hypoxic and normoxic conditions. In situations where endogenous H_2_O_2_ is limited, some nanoparticles can generate oxygen through water splitting. Nanocomposites based on carbon nitride and tungsten nitride nanoparticles can split water when exposed to light and thus increase the oxygen content. In addition, artificial oxygen carriers, such as nanocarriers loaded with perfluorocarbon or haemoglobin-based systems, can deliver molecular oxygen directly to tumor sites [144,145,146].

### 5.2. Regulating the Tumor Microenvironment (TME) to Increase Oxygen

Beyond direct oxygen generation, nanoparticles can modulate the TME to improve oxygen delivery. This includes strategies to increase blood flow, such as the use of nanomaterials that release nitric oxide or induce mild photothermal heating to improve tumor blood flow and oxygenation. Another approach is to reduce oxygen consumption within the TME by inhibiting oxidative respiration. One example of this is the concept of the “O_2_ economiser”, which aims to save endogenous O_2_ for PDT. Overcoming tumor hypoxia is not just about improving the efficacy of PDT; it is a critical factor for many oxygen-dependent cancer therapies, including radiotherapy. Nanoparticle strategies targeting hypoxia have wider implications for sensitising tumors to a range of treatments and could become a fundamental component of multimodal cancer therapy [147,148,149].

Another major challenge for PDT is that visible light only penetrates about 5–10 mm into the tissue, so it is mainly effective on tumors close to the surface. To solve this problem, researchers have moved from using external light sources to more invasive internal methods and even the use of other forms of energy. These advances are changing the way PDT can be used to treat deeper tissues [150].

### 5.3. NIR-Activated Nanoparticles

Near-infrared-activated nanoparticles work with NIR, which penetrates deeper into the tissue than visible light. For example, quantum dots can absorb NIR light and then emit light at specific wavelengths, allowing them to reach deeper tumors. In combination with NIR light, certain quantum dots can boost ROS production to treat lung cancer. Up-conversion nanoparticles are also important as they absorb NIR light and emit visible light, which then activates nearby photosensitisers in deeper tissues. In this method, continuous lasers with lower energy can be used, allowing the treatment of larger areas compared to two-photon PDT, which requires pulsed lasers and is limited to small areas [151,152,153].

### 5.4. Novel Light Delivery Systems and Alternative Energy Sources for Deep Activation

Researchers are also developing new ways to deliver light directly into tumors. Some use optical fibres with special tips to illuminate the surface or surrounding areas. In some cases, the fibers are inserted directly into the tumor to treat larger or deeper tumors. Spreading the light dose over several shorter sessions can also be beneficial, as the tissue has time to regenerate between treatments. Another important step forward is the search for ways to activate photosensitisers without the need for direct visible light. Attempts to circumvent the need for direct visible light rely on alternative activation sources such as X-rays or internal radiative processes; however, these approaches remain experimental and are not substitutes for conventional light-based PDT [154,155].

### 5.5. X-Ray-Activated Photodynamic Therapy (X-PDT)

This innovative strategy utilises low energy X-rays to excite photosensitizers via scintillating nanoparticle probes (nanoscintillators) that convert X-ray energy into visible or near-visible light to generate ROS in deep tissues that are otherwise inaccessible to classical PDT. This approach aims to ensure efficiency and accuracy while minimising damage to surrounding healthy tissue [156].

### 5.6. Cerenkov Radiation-Activated PDT

Cherenkov radiation is the characteristic blue glow that is generated during radiotherapy. Cerenkov radiation is low in intensity and is broadband and biased toward the ultraviolet–blue region, so its efficiency depends on matching the emission spectrum to the absorption of the photosensitizer and on the local radiation dose. Current reports suggest that while Cerenkov-driven PDT is feasible in preclinical models, further work is needed to determine whether sufficient light intensity can be achieved safely in patients [157]. In support of this, Guo et al. demonstrated that, after excluding the effects of radiotherapy and drug toxicity, Cerenkov radiation generated by high-energy γ-rays was able to activate the photosensitizer MPPa in A549 human lung carcinoma cells. Their results suggest that this internally generated light may help circumvent the penetration limits of traditional optical PDT and could potentially serve as a complementary strategy for treating deep-seated lesions [158]. This approach directly overcomes the penetration limitations of optical photon-based PDT and makes it a viable and complementary cancer therapy for deep-seated tumors. X-ray-activated photodynamic therapy and Cerenkov-activated PDT change the way light is used in PDT. By activating photosensitisers without direct visible light, these new methods could make PDT usable for many more types of deep-seated, hard-to-reach cancers that were previously untreatable [159].

## 6. The Role of the Tumor Microenvironment (TME) in Nanoparticle-Ehanced PDT

The tumor microenvironment (TME) is more than just a background for cancer progression. It is a complex and dynamic system that significantly influences how tumors grow, spread and respond to treatment. Features such as low oxygen levels, acidic pH, a stiff ECM and immunosuppression make it difficult for many cancer therapies to be effective. However, these same characteristics also create new opportunities for targeted treatments with nanoparticles [160,161].

The many factors within the TME make it difficult to treat cancer effectively. Low oxygen levels, common in solid tumors, limit the success of PDT as less oxygen will generate less ROS. This also contributes to tumors resisting treatment and evading the immune system [127,141,162]. The acidic environment in tumors caused by increased anaerobic glycolysis can weaken immune cells and disrupt the extracellular matrix. The stiffness of the extracellular matrix, which is often observed in solid tumors, hinders the transport of drugs to the cancer cells This stiffness promotes tumor growth, spread and drug resistance and even helps the tumors to excrete chemotherapeutic drugs. The TME also contains many immunosuppressive cells, which makes the effect of immunotherapy more difficult [163,164].

Nanoparticles offer new approaches to overcome barriers in the TME and can even alter the environment to improve the effectiveness of treatments. Instead of just delivering drugs, these smart nanoparticles can reshape the tumor’s environment to support therapy and fight the cancer. This approach is important for overcoming drug resistance and improving the effectiveness of treatments [55,127].

### 6.1. pH-Responsive Nanoparticles

These nanoparticles are designed to selectively release their therapeutic payloads in acidic TME, minimizing off-target effects on healthy tissue. One example is pH-responsive aerobic nanoparticles consisting of catalase and chitosan encapsulating chlorin e6. These nanoparticles break down in the acidic tumor environment and rapidly release catalase to generate oxygen in situ from endogenous H_2_O_2_, significantly increasing PDT efficacy in hypoxic tumors. Some pH-responsive nanoparticles can also neutralize excess acid, which can inhibit tumor growth and improve the efficacy of other therapeutics [165,166].

### 6.2. Enzyme-Responsive Nanoparticles

The unique enzymatic environment of tumors, which is characterized by the overexpression of certain enzymes, can be exploited by enzyme-responsive materials. These smart materials are designed to react with specific, highly expressed enzymes in tumor tissues, ensuring the precise release and delivery of photosensitizers at the target site. Examples include hyaluronidase-responsive materials, matrix metalloproteinase-responsive hydrogels, carboxylesterase-responsive materials, cathepsin B-responsive materials, and cytochrome P450-responsive nanoparticles. Furthermore, nanozymes are nanomaterials with enzyme-like catalytic activity that can produce ROS, regulate intracellular redox homeostasis, induce ferroptosis (a form of iron-dependent cell death), and enhance the anticancer effects of PDT when used in combination [167,168,169].

### 6.3. Immune Cell Infiltration Modulation

Photodynamic therapy itself stimulates the immune system, recruits neutrophils and macrophages and activates T helper and cytotoxic T lymphocytes. Photodynamic therapy enhanced with nanoparticles refines this even further. Porphyrin-cholesterol conjugates induce immunogenic cell death, which stimulates antigen-presenting cells to mature and activate cytotoxic CD8+ T cells, sensitizing cancer cells to immune checkpoint inhibitors. Pyrolipid-loaded Zn pyrophosphate (ZnP nanoparticles (ZnP@pyro)) have been shown to sensitize tumors to checkpoint inhibition by PD-L1 antibodies, leading to eradication of primary tumors and prevention of metastases [170]. Biodegradable mesoporous silica nanoparticles in combination with photosensitizing compound, neoantigen peptides and cytosine–guanine oligodeoxynucleotide adjuvant enable PET-guided PDT neoantigen-based cancer vaccination. This process, termed immuno-PDT, uses PDT-induced immunogenic cell death to convert an immune-off tumor into an immune-on tumor, effectively overcoming the intrinsic immunosuppressive tumor environment. Nanoparticles can also directly reprogram immunosuppressive tumor environment from an M2-like phenotype to an immunosuppressive M1-like phenotype or activate immunostimulatory tumor cells such as dendritic cells [171,172,173]. A new nano-immunotherapy uses nanoparticles loaded with anticancer drugs and dual antibodies (e.g., against CD47 and PD-L1) to deliver drugs directly to cancer cells while enhancing both innate and adaptive immune responses and minimizing toxicities. This suggests that PDT, especially in combination with nanotechnology, is becoming an effective immunomodulatory agent. It is not just about killing cells directly, but harnessing the body’s own defenses to achieve long-term control and prevent metastasis. This positions nanoparticle-based PDT as a cornerstone for future immuno-oncology strategies that may make resistant tumors susceptible to immune checkpoint inhibitors [174,175].

### 6.4. Extracellular Matrix Degradation

The dense and stiff ECM represents a physical barrier to drug release. Nanoparticles offer innovative strategies to modulate the tumor ECM by selectively targeting and degrading its components, such as collagen and hyaluronic acid, to improve drug penetration and distribution. These may include collagenase functionalized nanoparticles or strategies targeting cancer-associated fibroblasts, which play a critical role in mediating immunosuppression and promoting tumor progression by breaching the physical and biochemical barrier of the ECM [163,176].

## 7. Clinical Insights and Therapeutic Outcomes of PDT and Nanoparticle-Enhanced PDT

Photodynamic therapy (PDT) has been used in lung cancer management for quite some time, and over the years one thing has become very clear: the treatment works best when the right patients are selected and when the clinical protocol is followed carefully from start to finish. In day-to-day practice, the most consistent results come from treating early-stage disease, especially lesions that sit centrally in the airways. Carcinoma *in situ* (CIS) and small T1 tumors tend to respond particularly well because they usually spread along the airway surface and can be reached easily with bronchoscopic light delivery—still the cornerstone of effective PDT [177].

Clinicians often turn to PDT in a few specific situations: patients who are experiencing airway obstruction, those who would face high surgical risk, or individuals for whom preserving airway structure is especially important. Tumors that arise in the trachea, main bronchi, or lobar bronchi generally behave more predictably under PDT than peripheral nodules. Although CT-guided delivery has made treatment of certain peripheral lesions possible, central airway tumors remain the most suitable and well-studied candidates [178]. From a histologic standpoint, squamous cell carcinoma stands out as the subtype that responds most reliably, largely because of its tendency to grow along the mucosal surface. Some carefully chosen adenocarcinomas may also benefit, although the evidence is more limited [177].

The procedural steps used in most centers follow a familiar pattern. Treatment begins with the photosensitizer—porfimer sodium remains the workhorse agent—administered intravenously at 2.0 mg/kg. A waiting period of roughly 48 h is then observed so that the drug can accumulate in the tumor while background uptake in normal tissue declines [177]. The therapeutic bronchoscopy that follows involves delivering 630 nm light through optical fibers, sometimes tipped with a cylindrical diffuser when circumferential exposure is needed. Illumination times typically fall between 300 and 500 s, depending on the size and thickness of the lesion. Fluence levels are usually around 200 J/cm^2^ when using a diffuser and closer to 400 J/cm^2^ for direct-contact fibers [179].

Care after illumination is an important part of the protocol. A second bronchoscopy—usually performed 2 to 3 days later—is used to clear necrotic debris, assess airway patency, and decide whether additional PDT sessions are needed [177]. As with any interventional technique, complications can occur, so patient selection, cautious planning, and attentive follow-up care all play essential roles [178].

Table 2 shows a summary of the current clinical practice.

Photodynamic therapy has shown encouraging results in the treatment of certain types of lung cancer, and researchers are working to expand its use. Evaluation of clinical trials conducted since 2000 has shown consistently positive results for PDT in lung cancer [8,12].

In early stage lung cancer, PDT is becoming a standard option for the treatment of early superficial squamous cell carcinoma, peripheral-type lung cancer and microinvasive, radiographically occult lung cancer. In early central lung cancer, defined as carcinoma in situ or limited invasion, PDT has shown high complete response rates up to 100% in some lesions. The treatment is most effective for flat, small and superficial tumors. Tools such as white light bronchoscopy, autofluorescence bronchoscopy, endobronchial ultrasound and optical coherence tomography help to confirm tumor margins and improve treatment outcomes. PDT can also help make some inoperable patients eligible for surgery and allow surgeons to remove the tumor more completely. The fluorescence of photosensitizer activation helps verify whether tumor tissue is still present [180,181,182].

For patients with advanced lung cancer who cannot undergo surgery, PDT is a promising treatment. Smaller tumors respond better, with higher remission rates, especially if the cancer is confined to the mucosa. Photodynamic therapy can also improve the physical condition of patients, making them better candidates for further treatment [37,180].

Photodynamic therapy is increasingly being used for peripheral lung cancers by delivering fibers directly into the tumor, which helps fight the cancer while sparing healthy tissue. New tools such as advanced fiberscopes and robotic bronchoscopy are improving accuracy. CT-guided PDT has shown promising results, but there are still other issues, such as fiber movement and risks like pneumothorax and bleeding [183].

Patients with SCLC may benefit from PDT in a similar way to patients with NSCLC, so this treatment should be considered for both types [180].

The ability of PDT to elicit a robust immune response contributes to “long-lasting anti-tumor activity” that is instrumental in controlling metastasis and preventing recurrence [184].

Long-term follow-up studies of PDT for early central lung cancer have shown impressive 5- and 10-year overall survival rates (OS) of over 80% and 70%, respectively, for lesions ≤ 1.0 cm. For unresectable stage Tis and T1 patients, 5-year OS rates of 67.0% and 37.5%, respectively, have been reported [185,186].

Patients who achieve a fully reopened bronchial lumen after PDT have a significantly longer median OS (24.1 months vs. 14.9 months) and disease-free survival, 34.1 months vs. 7.5 months) than patients with incomplete reopening. A 14-year study found that patients with stage I disease treated with PDT had a 5-year survival rate of 93, while patients with stage II, IIIa, IIIb and IV disease had a median survival of 22.5, 5.7, 5.5 and 5.0 months, respectively [182,186].

The consistent finding that PDT is effective for specific conditions. However, most lung cancer cases involve larger, peripheral, advanced-stage tumors, which limits the standalone use of PDT. While success in these specific areas provides a strong foundation, the true transformative potential lies in expanding PDT’s applicability to the broader spectrum of lung cancer cases, where current treatments are often insufficient. This highlights the importance of ongoing research into overcoming limitations related to light penetration and hypoxia [187].

Photodynamic therapy is generally safe and effective at targeting tumors and often causes fewer side effects than traditional treatments. The primary complications associated with PDT using porfimer sodium are photosensitivity and hypoxia [187,188]. Photosensitivity, caused by the accumulation of the photosensitizer in the skin, can last for several months and requires patients to avoid direct and prolonged sunlight exposure. However, this is widely regarded as a minor side effect compared to the systemic toxicity associated with chemotherapy and radiotherapy. Hypoxia can occur 12–24 h after the procedure due to bronchial obstruction caused by tissue debris, inflammation, or edema. Non-specific uptake of photosensitizers in highly vascularized lung tissue can lead to the destruction of healthy tissue [12,189]. Short-term complications may include tumor sloughing and swelling of the surrounding normal tissue, which can lead to symptoms such as cough, hemoptysis, chest pain, and respiratory distress. Patients with advanced-stage disease are more likely to experience respiratory failure, infection, and hemoptysis. Perforation is rare, but pneumothorax may occur in patients with peripheral lesions or severe chronic obstructive pulmonary diseas. Drug allergic reactions are typically ruled out by a skin test. Long-term complications are generally mild [190]. The major concern is skin photosensitivity, which affects 3% to 31% of patients. Its duration varies with the photosensitizer used (e.g., 4 to 6 weeks for photofrin-PDT and 2 to 3 weeks for mono-L-aspartyl chlorin e6-PDT). Mild and transient infection and fever, rare fistula formation, and localized scarring and fibrosis may also occur. Notably, a study on early NSCLC reported no severe early or late PDT-related adverse events [191].

Traditional lung cancer treatments can cause resistance and unwanted side effects, and surgery is often risky. In contrast, the main side effect of PDT, photosensitivity, is usually mild. PDT is less invasive, more precisely targets tumors, and rarely leads to resistance. It also causes fewer long-term problems than chemotherapy. Combining PDT with nanoparticles could make the treatment safer and more effective, potentially improving patients’ quality of life and enabling more frequent treatments [8,180].

Although conventional PDT is well-established, the use of nanoparticles with PDT is still primarily being tested in laboratories and early studies. These new systems have demonstrated strong anti-cancer effects in preclinical research. However, there is currently insufficient clinical data. More clinical trials are needed to confirm these early, promising results [12,13,42].

## 8. Synergistic Combination Therapies with Nanoparticle-Enhanced PDT

Because lung cancer is complex and varies widely, treating it often requires more than one type of therapy. Using nanoparticles with PDT allows different cancer treatments to be combined in a way that leverages their strengths and addresses the limitations of using a single method. This approach marks a shift toward more integrated strategies where nanoparticles facilitate the simultaneous delivery and activation of multiple treatments. The goal is to more effectively target lung cancer, reduce the likelihood of drug resistance, and improve long-term outcomes [11,12,13,189].

### 8.1. Combination with Chemotherapy

Using nanomaterials to combine PDT with chemotherapy offers several benefits, including improved drug delivery, reduced harm to healthy tissues, and enhanced treatment effects. Nanoparticles can deliver chemotherapy drugs directly to tumor cells, increasing the drug concentration where it is needed and reducing side effects. This combination can make treatment more effective by using the different ways PDT and chemotherapy attack cancer [12,13,55].

For example, a nanodrug delivery system combining aminated mesoporous graphene oxide, protoporphyrin IX, and hyaluronic acid loaded with osimertinib demonstrated significant efficacy in in vivo and in vitro models. This system leverages the photothermal conversion properties of mesoporous graphene oxide and the photosensitizing effects of protoporphyrin IX to generate ROS under laser irradiation, thereby enhancing therapeutic effects against NSCLC [192].

In another study, researchers combined hypocrellin B, which was encapsulated in hyaluronic acid-ceramide nanoparticles, with paclitaxel, an anticancer agent. The Hypocrellin B-paclitaxel nanoparticles treatment group exhibited the most effective anti-cancer effects in human lung adenocarcinoma cells and A549 tumor-bearing mice [193,194].

Polydopamine/Au hollow nanospheres loaded with doxorubicin demonstrated significant anti-tumor and anti-metastatic effects in NSCLC models by combining the benefits of chemotherapy and photothermal therapy [195].

### 8.2. Combination with Immunotherapy

Immune checkpoint inhibitors, such as those targeting programmed cell death protein 1 or programmed death-ligand 1 (PD-L1) have helped lung cancer patients live longer. However, their effectiveness is often limited by the tumor’s ability to suppress the immune system. Using nanoparticles with PDT can help by causing a type of cell death that alerts and activates the immune system at the tumor site. This process helps immune cells recognize and attack cancer cells, making them more responsive to immune checkpoint inhibitors. Consequently, tumors that were previously unresponsive to immunotherapy may become sensitive, resulting in a stronger, longer-lasting immune response that could prevent the cancer from recurring [196,197,198].

For example, porphyrin cholesterol conjugates have been designed for synergistic PDT-immunotherapy in lung cancer, inducing immunogenic cell death and activating T cells [199].

Zinc pyrophosphate loaded with the photosensitizer pyrolipid have been shown to sensitize tumors to PD-L1 antibody-mediated checkpoint inhibition, eradicating the primary tumor and preventing metastasis [200].

Biodegradable mesoporous silica nanoparticles loaded with photosensitizer chlorin e6, neoantigen peptides, and cytosine–guanine oligodeoxynucleotide adjuvant have enabled positron emission tomography-guided PDT neoantigen-based cancer vaccination. This treatment has elicited strong antitumor immune responses and effectively treated both local and distant tumors [173].

A newly developed nano-immunotherapy uses nanoparticles loaded with anti-cancer drugs and dual-targeting antibodies (e.g., against CD47 and PD-L1) to deliver drugs directly to cancer cells. This method simultaneously boosts innate and adaptive immune responses while minimizing toxicities [122,175].

### 8.3. Potential Synergies with Targeted Therapy, Radiation Therapy, and Gene Therapy

While the literature primarily details combinations with chemotherapy and immunotherapy, the principles of nanoparticle-enhanced PDT suggest strong potential for synergy with other cancer therapies [201].

Targeted therapy drugs can be delivered directly to cancer cells using nanoparticles, increasing the drug concentration at the tumor site while reducing side effects elsewhere in the body. Photodynemic therapy can trigger various mechanisms of cell death, which may help overcome the resistance that often develops with single-targeted drugs. For instance, gold nanoparticles paired with antibodies that recognize lung cancer stem cells can improve photosensitizers’ ability to reach and remain in these cells, thereby enhancing the effectiveness of PDT. This method specifically targets cancer cells, does not suppress the immune system, and is less likely to induce resistance [55,61].

Some nanoparticles, especially those made from gold or other metals, can increase the effectiveness of radiation therapy by helping it target cancer cells more precisely. Combining them with PDT could create a powerful two-part treatment. This combination may allow doctors to use lower radiation doses, thereby protecting healthy tissue. Some nanoparticles can also produce oxygen inside tumors, making radiation therapy more effective. Another new idea is using X-rays to activate PDT deep inside the body, which could help treat tumors that are difficult to reach with light [13,202,203,204].

Nanoparticles are often used to deliver gene therapy materials, such as therapeutic genes or small interfering RNAs (siRNAs), directly into cancer cells. When combined with PDT, these nanoparticles can deliver the photosensitizer and genetic material simultaneously. The stress caused by PDT may enhance the effectiveness of gene therapy by increasing the likelihood that cells will take up or respond to the treatment. Some nanoparticles can be designed to release their contents only at the tumor site, making the treatment more targeted. For example, liposomes and other nanoparticles have been used to deliver siRNA to lung cancer cells, resulting in improved tumor shrinkage and growth control [205,206].

## 9. Challenges and Future Directions for Clinical Translation

Nanoparticle-enhanced PDT shows great promise for treating lung cancer. However, moving from early research to routine clinical use faces many obstacles. These include complex regulations, manufacturing and cost issues, the need for personalized treatments, and important safety and ethical questions [11,13,204].

One of the biggest issues is the shallow penetration of visible light. Because the light does not travel far into tissue, PDT mainly works on tumors that lie close to the airway surface, which unfortunately leaves many peripheral lesions out of reach [207,208]. Tumor hypoxia adds another layer of difficulty. Many lung cancers contain areas with poor oxygen levels, and since PDT depends on oxygen to generate reactive oxygen species (ROS), this can seriously weaken its therapeutic effect [208,209].

Another practical challenge is the inconsistent uptake of photosensitizers. The amount of drug that reaches the tumor can vary widely between patients—and even between different regions of the same tumor—making treatment responses hard to predict [146,208]. On top of that, PDT requires bronchoscopic access, which naturally limits its use to centrally located airway tumors and narrows the pool of suitable patients [210]. Although several emerging strategies show promise, most remain at an early stage, and additional evidence will be required before any can be considered clinically feasible.

Despite these hurdles, ongoing research is actively trying to refine and extend the role of PDT. A large portion of this work focuses on nanoparticle-based delivery systems designed to improve how evenly photosensitizers are distributed throughout the tumor and to boost overall uptake [211]. Other groups are looking at materials capable of storing or releasing oxygen, hoping to counteract the effects of hypoxia and make PDT more reliable in oxygen-poor environments [212]. PDT has clear limitations and is not appropriate for systemic malignancies such as leukemia or for deep organs that accumulate photosensitizers but cannot be safely illuminated, including liver and spleen.

There is also growing interest in activation strategies that sidestep the light-penetration problem altogether. Approaches using near-infrared (NIR) light, upconversion nanoparticles, and even X-rays or Cerenkov radiation are being explored as ways to reach deeper tissue layers and expand PDT beyond its traditional limits [207,213].

### 9.1. Regulatory Considerations and Approval Pathways

Regulating nanomedicine is challenging because nanoparticle therapies do not fit neatly into existing categories, such as drugs or devices. Their unique features and manufacturing processes can alter their behavior, complicating the approval process. Since there are no clear guidelines, it is important to work closely with agencies like the FDA and design thorough clinical trials. For lung cancer, the FDA typically considers OS as the primary measure for approval. Other measures, such as time to progression or objective tumor response rates, can facilitate faster approvals, but they do not always predict survival unless the results are strong and sustained. Photodynamic therapy has gained approval in some cases when it has clearly improved patient symptoms. However, measuring tumor outcomes can be subjective. There is a need for better patient-reported outcome tools and clearer trial designs [63,214,215].

### 9.2. Manufacturing Scalability and Quality Control

A significant hurdle in the clinical translation of nanomedicines is achieving reproducible manufacturing and large-scale production while ensuring stability under physiological conditions. The intricate processes involved in synthesizing complex nanoparticle structures, such as layer-by-layer nanoparticles, make large-scale production difficult and affect repeatability and quality control. Achieving biostability within the physiological environment and active drug targeting without off-target accumulation remain persistent challenges. The high cost of raw materials and the complexity of manufacturing processes further hinder cost-effective, large-scale production [16,216]. To address these issues, it is essential to implement continuous production methods, such as microfluidics, and real-time monitoring systems to maintain consistency and quality assurance. Compliance with good manufacturing practice standards, including rigorous process validation and stability testing, is crucial for ensuring the safety and reliability of clinical applications. These challenges highlight a significant “valley of death” in translating promising nanomedicine research into clinical reality. Overcoming this requires scientific breakthroughs, regulatory harmonization, industrial-scale innovation, and robust long-term safety profiling [217,218].

### 9.3. Cost-Effectiveness Analysis

Photodynamic therapy has been shown to be more cost-effective than surgery for early-stage lung cancer. One study found that total and monthly treatment costs were lower for PDT than for surgery, particularly for small tumors. However, the cost benefits of standard PDT could decrease if advanced nanoparticles are costly to produce. High prices could also discourage companies from developing these therapies [12].

### 9.4. Development of Personalized Treatment Approaches

Personalized medicine, with therapies tailored to each patient’s tumor features, is the future of cancer treatment. Combining artificial intelligence (AI) with nanomedicine could transform this field. Artificial intelligence can analyze large amounts of data to help design treatment plans and predict how patients will respond to nanoparticle therapies. This assists in selecting the most effective drug combinations and nanoparticle types, which is particularly beneficial for treating complex tumors and preventing drug resistance. Artificial intelligence can also improve nanoparticle design, identify new biomarkers, and monitor treatment in real time. Wearable devices with AI can monitor patients for early signs of complications. Artificial intelligence can also help overcome challenges in measuring and delivering the correct PDT dose. Overall, AI could make personalized, nanoparticle-based PDT more practical and effective in clinical settings [44,219,220,221].

### 9.5. Long-Term Safety, Immunogenicity, and Ethical Consideration

The long-term safety of nanoparticle-based cancer therapies is a significant concern. Certain nanoparticles, especially those made from heavy metals (e.g., gold, silver, and quantum dots), can accumulate in organs such as the liver, kidneys, and spleen, potentially causing long-term toxicity. Even biodegradable nanoparticles can generate toxic degradation products. Comprehensive toxicity studies and long-term monitoring are essential to mitigate these risks [222].

Another important issue is how the immune system reacts to nanoparticles. Some types, especially those that do not break down, can trigger immune responses such as inflammation or allergic reactions. PEGylated nanoparticles, for example, have been shown to cause such effects. When proteins bind to nanoparticles in the blood, it can alter how the body perceives them, leading to their removal. It is also challenging to combine immune-modulating drugs with nanoparticles while maintaining their effectiveness [223,224].

Ethical considerations in nanomedicine include risk assessment, the distinction between somatic and germline cell therapy, human enhancement, stem cell research, toxicity, uncontrolled function, and self-assembly. The unique properties of nanoscale materials introduce complexity, but the core principles of medical ethics—respect, beneficence, nonmaleficence, and fairness—remain applicable. Careful weighing of benefits and risks during development and use remains essential [225].

## 10. Conclusions

Lung cancer is a major global health problem. It often develops quickly, is usually diagnosed late, and occurs frequently. Although standard treatments are important, they often cause significant side effects and can become less effective over time. This underscores the need for more targeted and less harmful therapies. Photodynamic therapy is a less invasive option that works by allowing photosensitizers to build up in tumor tissue. When exposed to light, these agents produce ROS that kill cancer cells. Photodynamic therapy can destroy tumor cells directly, damage blood vessels that support tumors, and help the immune system fight cancer. This makes PDT a flexible tool in cancer treatment [226].

Incorporating nanotechnology has significantly improved the efficacy of PDT. Nanoparticles function as advanced delivery systems, overcoming limitations of traditional photosensitizers, such as low solubility, non-specific distribution, and inadequate tumor accumulation. Engineering the size, shape, and surface properties of nanoparticles enables both passive targeting through the enhanced EPR effect and active targeting via ligand-receptor interactions. This approach increases the concentration of photosensitizers at the tumor site while reducing systemic exposure and toxicity [12,227].

Nanoparticle technology addresses key challenges in PDT, such as low oxygen levels in tumors and limited light penetration. New types of nanoparticles can generate oxygen or alter the tumor environment, thereby enhancing the effectiveness of PDT. Meanwhile, researchers are developing nanoparticles that respond to NIR light and new light delivery methods, such as tiny implanted lights or fibers. There are also new methods that use X-rays or Cerenkov radiation to activate PDT, which enables the treatment of tumors located deep within the body. These advances are transforming PDT from a treatment for surface tumors into a treatment for many types of cancer [13,228,229].

The TME’s factors such as hypoxia, acid pH level, ECM rigidity and immune suppresion hinder effective cancer therapy. Not only are nanoparticles overcoming these obstacles, they are also actively modifying the TME. pH-responsive and enzyme-responsive nanoparticles facilitate targeted drug release within the tumor’s specific biochemical environment. Additionally, advanced nano-immunotherapy approaches aim to reprogram immunosuppressive immune cells and convert immunologically unresponsive tumors into responsive ones, thereby improving immunotherapy outcomes. While ongoing research aims to minimize limitations such as hypoxia, limited light penetration, and heterogeneous biodistribution, these constraints are intrinsic to PDT and may never be completely eliminated [127,169,230].

Using nanoparticle-enhanced PDT alongside other cancer treatments shows considerable promise. When combined with chemotherapy, PDT can deliver drugs more effectively and improve treatment outcomes. When combined with immunotherapy, nanoparticle-enhanced PDT can make cancer cells more visible to the immune system, thereby enhancing the effectiveness of other treatments and leading to longer-lasting responses. Recent research also suggests that combining PDT with targeted therapy, radiation therapy, or gene therapy could be beneficial. These approaches involve combining multiple treatments to overcome resistance and control tumors more effectively [12,13,40,231].

Despite these advances, incorporating nanoparticle-enhanced PDT into everyday medical practice remains challenging. Because nanomedicine is complex, clear rules and strong safety data are needed. It is also difficult to produce these treatments on a large scale, maintain batch consistency, and ensure affordability. Long-term studies are necessary to ensure their safety and determine whether they cause unwanted immune reactions. There are also important ethical questions that require careful consideration [73,204].

Advancing nanoparticle-enhanced PDT for lung cancer requires innovative ideas and collaboration across various fields. Using AI to integrate imaging (radiomics), genomic, and clinical data may help identify predictive biomarkers for PDT response, guide nanoparticle design (e.g., choice of targeting ligands or payloads), and support treatment planning by forecasting which patients are most likely to benefit from nanoparticle-enhanced PDT. To make this therapy widely available and effective, ongoing research, clear regulations, large-scale production, and thorough clinical trials are necessary to ensure safe, accessible care for lung cancer patients worldwide [11,232,233].

Looking ahead, we believe that the most meaningful progress will come from bringing these different strands together rather than pursuing any single technology in isolation. In our view, the next generation of PDT for lung cancer will combine smarter nanocarriers, more reliable light-delivery solutions, and a much better understanding of each patient’s tumour biology, supported by modern imaging, genomics, and data-driven tools. If these elements can be integrated into pragmatic clinical trials and cost-conscious manufacturing pathways, PDT could move from a niche option for selected airway lesions to a genuinely versatile platform therapy. Our hope is that, within the next decade, nanoparticle-enhanced PDT will become part of personalised treatment algorithms for lung cancer, offering patients effective local control with fewer long-term toxicities and a better quality of life.

## Figures and Tables

**Table 1:** Key Properties and clinical status of photosensitizers in PDT.

Photosensitizer Name	Generation	Wavelength (nm)	Singlet Oxygen Yield	Key Properties	Clinical Use/ Approval	Major Limitations
Photofrin II (Porfimer sodium) [46]	1st	~630	Type II	Clinically established; deep but limited penetration; heterogeneous mixture	Lung, esophageal, bladder cancer	Prolonged photosensitivity, non-specific accumulation
Verteporfin (Visudyne) [47]	2nd	~690 (implied)	Type II	Liposomal formulation, rapid clearance	Age-related macular degeneration; under investigation for cholangiocarcinoma, ovarian cancer	Less photostable, susceptible to self-polymerization/photobleaching
5-aminolevulinic acid [48]	2nd	~635 (PpIX)	Type II	Endogenous conversion to PpIX; preferential accumulation in dysplastic tissue	Early-stage skin cancers, actinic keratosis; under investigation for bladder, head and neck cancers	Uneven distribution, limited laser penetration depth
Temoporfin (mTHPC, Foscan) [49]	2nd	~652	Type II	Strong absorption; high phototoxicity; long tissue retention	Head and neck, esophageal, gastric, gastrointestinal cancers	Marked photosensitivity; high lipophilicity; risk of off-target phototoxicity
Photocyanine [50]	2nd	~670	High	High quantum yield, low dark toxicity, minimal skin phototoxicity	Phase II clinical trials for various cancers including bronchial	Relatively shallow tissue penetration limits effectiveness

**Table 2:** Current clinical practice.

Category	Practical Points from Current Clinical Practice
Best-suited tumor stages	CIS and small T1 lesions; select superficial T2 lesions with limited mucosal involvement
Tumor location	Most reliable results in central airways (trachea, main bronchi, lobar bronchi); select peripheral lesions treated with CT-guided or navigational techniques
Histology	Strongest evidence for squamous cell carcinoma; certain superficially spreading adenocarcinomas may also be eligible
Ideal patient profile	Poor surgical candidates; patients with airway obstruction; those needing airway-preserving treatment rather than resection
Photosensitizer protocol	Porfimer sodium 2.0 mg/kg IV; ~48 h interval before illumination to improve tumor uptake and reduce background fluorescence
Light delivery	630 nm wavelength (Photofrin); optical fiber or diffuser tip depending on lesion shape; typical fluence ~200 J/cm^2^ (diffuser) or ~400 J/cm^2^ (contact)
Illumination timing	Usually 300–500 s per site, adjusted according to tumor size and depth
Post-procedure care	Bronchoscopy at 2–3 days for debridement and reassessment; repeat PDT if residual disease is present
Follow-up	Surveillance bronchoscopy at 4–6 weeks; imaging for deeper or peripheral lesions

## Data Availability

No new data were created or analyzed in this study.
